# A general semi-parametric approach to the analysis of genetic association studies in population-based designs

**DOI:** 10.1186/1471-2156-14-13

**Published:** 2013-02-28

**Authors:** Sharon Lutz, Wai-Ki Yip, John Hokanson, Nan Laird, Christoph Lange

**Affiliations:** 1 Department of Biostatistics, University of Colorado Anschutz Medical Campus, Aurora, USA; 2 Department of Epidemiology, University of Colorado Anschutz Medical Campus, Aurora, USA; 3 Department of Biostatistics, Harvard School of Public Health, Boston, USA; 4 Channing Laboratory, Harvard Medical School, Boston, USA; 5 Institute for Genomic Mathematics, University of Bonn, Bonn, Germany; 6, German Center for Neurodegenerative Diseases (DZNE), Bonn, Germany

**Keywords:** Genetic associations studies, Secondary phenotypes, Case-control, Ascertainment, Semi-parametric

## Abstract

**Background:**

For genetic association studies in designs of unrelated individuals, current statistical methodology typically models the phenotype of interest as a function of the genotype and assumes a known statistical model for the phenotype. In the analysis of complex phenotypes, especially in the presence of ascertainment conditions, the specification of such model assumptions is not straight-forward and is error-prone, potentially causing misleading results.

**Results:**

In this paper, we propose an alternative approach that treats the genotype as the random variable and conditions upon the phenotype. Thereby, the validity of the approach does not depend on the correctness of assumptions about the phenotypic model. Misspecification of the phenotypic model may lead to reduced statistical power. Theoretical derivations and simulation studies demonstrate both the validity and the advantages of the approach over existing methodology. In the COPDGene study (a GWAS for Chronic Obstructive Pulmonary Disease (COPD)), we apply the approach to a secondary, quantitative phenotype, the Fagerstrom nicotine dependence score, that is correlated with COPD affection status. The software package that implements this method is available.

**Conclusions:**

The flexibility of this approach enables the straight-forward application to quantitative phenotypes and binary traits in ascertained and unascertained samples. In addition to its robustness features, our method provides the platform for the construction of complex statistical models for longitudinal data, multivariate data, multi-marker tests, rare-variant analysis, and others.

## Background

In genetic association studies, individuals are often recruited based on case-control ascertainment conditions of the primary phenotype [1]. For the analysis of secondary phenotypes, this recruitment-scheme can become problematic. If the secondary phenotype is correlated with the primary phenotype in a case-control study, the distribution of the secondary phenotype can be fundamentally different from the general population. For example, in a genetic association study of COPD in which all cases have COPD and control subjects have normal pulmonary function, the distribution of quantitative lung phenotypes can deviate substantially from their distribution in the general population. For samples that are ascertained in this fashion, standard statistical methods may lead to misleading results or may lack statistical power to identify true genotype phenotype associations. There are several methods to accurately estimate the odds ratio of genetic variants for binary secondary phenotypes associated with case-control status [2-10], but these methods cannot easily accommodate continuous secondary phenotypes. For the special case that the secondary phenotype is normally distributed or binary, Lin & Zeng (2009) proposed an adjusted score test that incorporates genetic associations with affection status into the test statistic [11].

We present a more general approach that does not require any distribution assumptions for the secondary phenotype. We refer to the approach as the non-parametric population-based association test (NPBAT). The approach has a form similar to the Family Based Association Test (FBAT), a non-parametric test statistic that is frequently used in the family based setting [12-15]. The flexibility of our approach allows us to construct a genetic association test for standard and complex phenotypes that is non-parametric with respect to the phenotype. The class of tests is very general. It includes most standard association tests and can be applied to multivariate traits and phenotypes, multiple genetic markers, and case- control studies where phenotypic information is available for the cases but correlated with the case-control status [16-18].

The general concept of the proposed association-testing framework is to condition on the phenotype of interest and treat only the genetic data as random [12,13,15]. By assuming that the phenotype data is deterministic, the validity of the approach does not depend on the correctness of the phenotypic assumptions. Nevertheless, the power of the approach can be increased by incorporating a plausible model for the phenotype into the test statistic. Based on theoretical considerations and on simulation studies, we show that the new approach is robust against misspecification of phenotype assumptions. At the same time, this approach achieves the same power level as standard genetic association tests for population-based designs when the phenotype of interest has a normal distribution or is dichotomous. For studies where a quantitative trait is correlated with case-control status, our simulation studies examine the power and significance levels for the proposed approach, which does not require any adjustment for the ascertainment conditions.

We illustrate the practical advantages of NPBAT by an application to the COPDGene study. The COPDGene study is a case-control study of the genetics of COPD in current or former smokers with at least 10 pack-years of smoking history [19]. We test the genetic association of single nucleotide polymorphisms (SNPs) in the CHRNA 3/5 region and the Fagerstrom Nicotine Dependence score (FNDS). FNDS is a validated instrument of nicotine dependence in current smokers and was measured in the current smokers, but not former smokers in the COPDGene study. NPBAT, which uses the genotype data in both current and former smokers, is compared to the published genetic association of SNPs in the CHRNA 3/5 region and FNDS that was performed in current smokers only [20].

## Methods

In a genetic association study, *n* unrelated study subjects have been recruited based on a predefined ascertainment condition. Let *X*_*i*_ denote the genotype of the individual *i*. The specific value of *X*_*i*_ will depend upon the genetic model under consideration. For instance, for an additive model, *X*_*i*_ = 0, 1, 2 for 0, 1, 2 disease alleles, respectively. *X*_*i*_ may also be a vector in order to test several alleles simultaneously. Let *T*_*i*_ denote the numerical trait information for individual *i*. For example, *T*_*i*_ could equal one for affected individuals and *T*_*i*_ could equal zero for unaffected individuals. Different coding functions are applied depending on the phenotype of interest. For binary and continuous traits, we will discuss efficient coding schemes below. First, we define a general class of test statistics as

(1) $$S=\overset{n}{\underset{i=1}{\sum }}\left(X_{i}-E_{x}\right)T_{i}$$

Note that *E*(*S*) = 0 under the null hypothesis of no association between the genotype *X* and the phenotype *Y*. Constructing a conditional score test in which the genotype *X*_*i*_ is the dependent variable and we condition upon the numerical trait information *T*_*i*_, the NPBAT statistic has the following form:

(2) $$\text{Sta}t_{\text{NPBAT}}=\frac{S-E[S]}{\sqrt{\text{var}(S)}}=\frac{\overset{n}{\underset{i=1}{\sum }}\left(X_{i}-E_{x}\right)T_{i}}{\sqrt{\left(\overset{n}{\underset{i=1}{\sum }}T_{i}^{2}\right)\left(\frac{\overset{n}{\underset{i=1}{\sum }}{\left(X_{i}-E_{x}\right)}^{2}}{n-1}\right)}}$$

where *E*_*x*_ denotes the expectation of the marker score/ genotype *X* under the null-hypothesis of no genetic association between the phenotype. The marker locus. *E*_*x*_ can be estimated based on the sample mean of the genotypes. The asymptotic distribution of the NPBAT statistic under the null-hypothesis depends on the estimation of *E*_*x*_ and on the specification of the trait information *T*_*i*_, and is derived in the Appendix.

There are various ways to code the phenotype of interest and define the coding function *T*_*i*_. For the analysis of affection status, one could specify the coding function to be *T*_*i*_ = 1 or *T*_*i*_ = 0, depending on the disease status of the proband. However, as we show in the Appendix Appendix A: Offset choice when Y is binary, a more efficient way is to set $T_{i}=1-\frac{\text{\#cases}}{n}$ for the cases, and $T_{i}=0-\frac{\text{\#cases}}{n}$ for the controls. Then the NPBAT statistic is approximately the same as the Cochran-Armitage Trend test.

If the phenotype *Y*_*i*_ is in fact normally distributed and $T_{i}=Y_{i}-{Ŷ}_{i}$ where ${Ŷ}_{i}$ denotes the fitted values of regressing the phenotype *Y* on any covariates, then the NPBAT statistic is approximately the same as a t-statistic from a linear regression. In general, if the phenotype *Y*_*i*_ is a continuous phenotype, we recommend *T*_*i*_ = *Y*_*i*_ - *μ*_*y*_ where *μ*_*y*_ is the phenotypic mean in the general population.

While it is appealing that the NPBAT statistic is comparable to standard methods in these simple scenarios, the real appeal of the NPBAT statistic is when there is only phenotype information available for some subjects but there is genetic information available for all subjects. For example, in case control studies, an additional quantitative phenotype may be available for the cases but not the controls. When testing for a genetic association with this additional quantitative phenotype, the NPBAT statistic uses the genotype of both the cases and the controls with the optimal coded phenotype *T*_*i*_ = *Y*_*i*_ - *Y*_offset_ where *Y*_offset_ is a constant. The choice of this constant is described in detail in the simulations sub-section and the asymptotic distribution of the NPBAT statistic is derived in the Appendix. Using this optimal offset choice, the NPBAT statistic has a substantial increase in power over other methods such as the NPBAT statistic when an offset choice of $T_{i}=Y_{i}-\bar{Y}$ or the improved score test, which is uniformly more powerful than score tests based on the generalized linear model such as the Cochran-Armitage trend test, the allelic *χ*^2^ test and the genotypic *χ*^2^ test [21].

### Adjustments for population admixture

The NPBAT statistic can be adjusted for population admixture by using standard methods such as principal components analysis or genomic control [22,23]. For example, to account for population admixture, one can treat the principal components as additional covariate representing population information, and incorporate them into the test statistic in equation (2) by taking $T_{i}=Y_{i}-{Ŷ}_{i}$ where ${Ŷ}_{i}$ denotes the fitted values of regressing the phenotype *Y* on the top principal components that explain the greatest amount of variability in the data. Note the above approach requires that the phenotype Y is dichotomous or roughly normally distributed.

### Extension to multiple phenotypes

The NPBAT statistic can be extended to *m* phenotypes to test the null hypothesis that a marker locus is not linked to any disease-susceptibility locus for any of *m* selected phenotypes. Then the test statistic becomes

(3) $$S=\overset{n}{\underset{i=1}{\sum }}(X_{i}-E_{x})T_{i}$$

Note that *E*(*S*) = 0 as is the case for the univariate version above. But here *T*_*i*_ is the *m* × 1 vector for the m phenotypes and *X*_*i*_ is just one marker. So S is *m* × 1. The *m* × *m* variance matrix is the following

(4) $$V_{S}=\hat{\sigma_{X}^{2}}\overset{n}{\underset{i=1}{\sum }}T_{i}T_{i}^{t}$$

where $\hat{\sigma_{X}^{2}}$ is the variance for marker X based on sample. Then the NPBAT statistic is the following

(5) $$\chi_{\text{NPBAT}}^{2}=S^{t}V_{S}^{-1}S$$

Due to the estimation of *E*_*x*_ based on the sample, this statistic does not have a chi square distribution and a permutation test needs to be used to assess significance levels, which can be done by using the NPBAT software package (https://sites.google.com/site/genenpbat/).

### Simulations

In genetic association case-control studies, only the cases may have additional phenotypic information available. For instance, in a case-control study where the cases have asthma (the primary phenotype), only the cases may have FEV measurements (the secondary phenotype). In this scenario, the secondary phenotype FEV will be more severe than it would be in the general population and the analysis of this secondary phenotype can be misleading due to the ascertainment of subjects based on the primary phenotype, asthma. To simulate this scenario, we generated the genotype X for 500 cases and 500 controls and a secondary phenotype Y for only the 500 cases from a truncated normal distribution with standard deviation *σ* = 1, mean *aX* under the alternative and mean 0 under the null and cutoff such that the secondary phenotype in the top 50 percent of the normal distribution. We consider an allele frequency of *p* = 20*%* and *a* is chosen such that the heritability *h*[24] equals 1*%*, 2*%*, 3*%*, 5*%*. The solving for a, $a=\sigma \sqrt{h/2p(1-p)(1-h)}$.

We compute the NPBAT statistic with the coded phenotype *T*_*i*_ = *Y*_*i*_ - *Y*_offset_ where *Y*_offset_ is a constant that ranges from -5 to 15 and *E*_*x*_ is the sample mean of the genotypes in the cases. We also compute the NPBAT statistic with *E*_*x*_ equal to the sample mean of the genotypes in the controls and *E*_*x*_ equal to the sample mean of the genotypes in the cases and the controls. We compare the power of these three NPBAT statistics to the Improved Score Test, which is uniformly more powerful than score tests based on the generalized linear model such as the Cochran-Armitage trend test, the allelic *χ*^2^ test and the genotypic *χ*^2^ test [21]. We also compare the power of the NPBAT approach to a standard linear regression.

**Figure 1:**
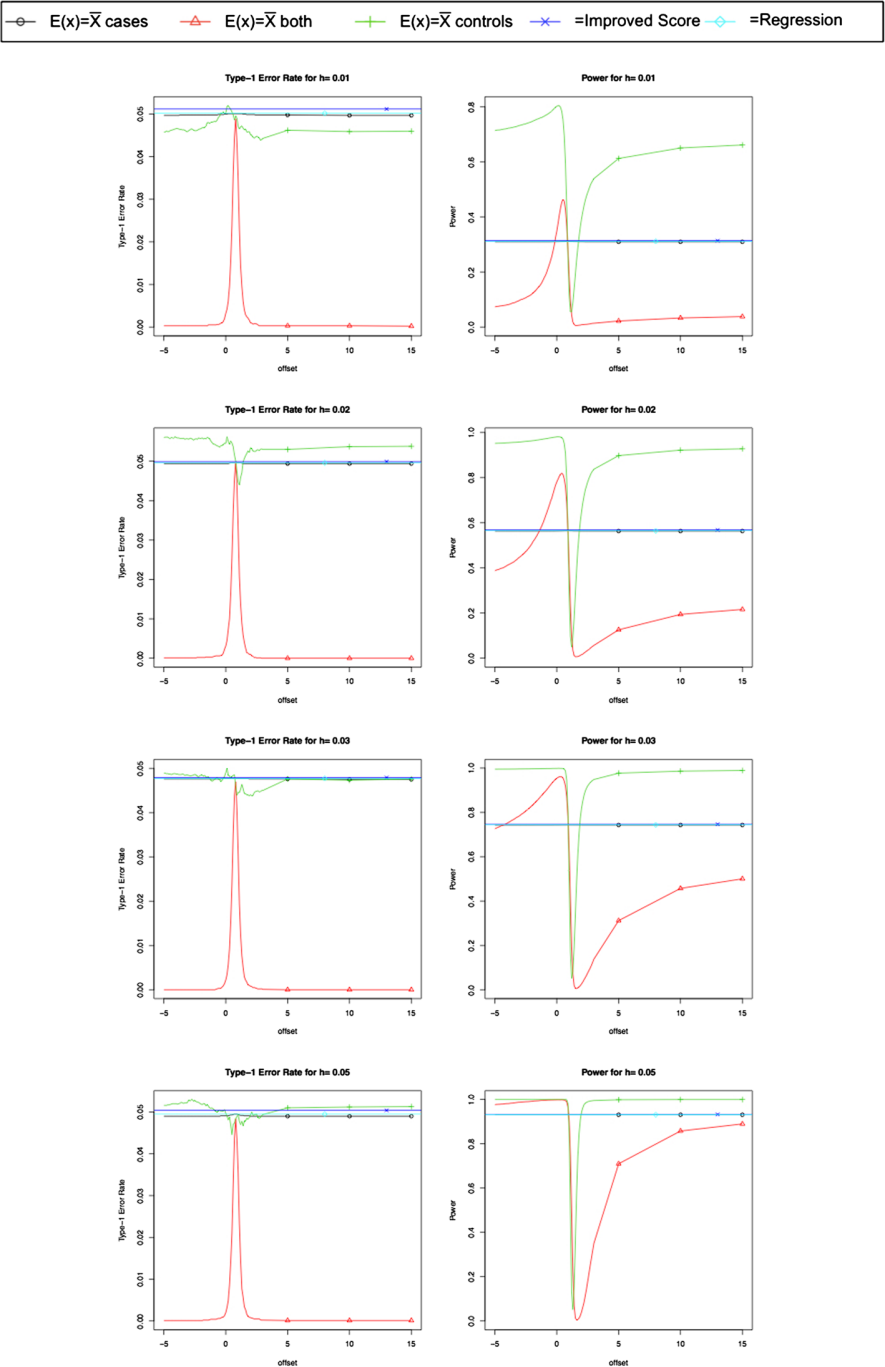
**Power and Significance levels for NPBAT, the Improved Score Test and the Likelihood Ratio Test (LRT).** This plot compares the power and type-1 error rate of the NPBAT method using *E*_*x*_ based on the sample mean of the cases, the controls and both the cases and controls. The power and significance levels of this method is compared to the improved score test and a standard linear regression. Note that the spike or drop in all the plots occurs where $Y_{\text{offset}}\approx \bar{Y}$, the sample mean of the secondary phenotype for the cases since the secondary phenotype is not available for the controls in this scenario. The power of the NPBAT approach is maximized when *E*_*x*_ is based on the genotype of the controls and *Y*_offset_ is significantly different than the phenotypic mean of the cases. When *E*_*x*_ is based on the genotype of the cases, the power of the NPBAT approach is similar to the improved score test and the regression. Note that the power of NPBAT approach when *E*_*x*_ is based on the genotype of both the cases and the controls is best for high values of heritability.

Under the null hypothesis, the NPBAT method maintains a significance level of approximately 5% or less as seen in Figure 1 whether *E*_*x*_ is the sample mean of the cases or the controls or both. Figure 1 also depicts the power results of these simulations. Note that the spike or drop in all the plots occurs where $Y_{\text{offset}}\approx \bar{Y}$, the sample mean of the secondary phenotype for the cases since the secondary phenotype is not available for the controls in this scenario. The power of the NPBAT approach is maximized when *E*_*x*_ is based on the genotype of the controls and *Y*_offset_ is significantly different than the phenotypic mean of the cases. When *E*_*x*_ is based on the genotype of the cases, the power of the NPBAT approach is similar to the improved score test and the regression. Note that the power of NPBAT approach when *E*_*x*_ is based on the genotype of both the cases and the controls is best for high values of heritability.

These simulations show that for case-control studies when analyzing secondary phenotypes correlated with case-control status, we recommend to set *Y*_offset_ to a constant significantly different from the phenotypic mean of the sample and *E*_*x*_ equal to the genotypic mean of the controls. In this situation, a robust and efficient choice for the offset *Y*_offset_ is the phenotypic mean in the general population. Note that the results of these simulations are analogous to the FBAT statistic in family studies where it was found that when ascertaining cases only from a quantitative distribution, one needed to choose an offset that was outside the range of the case’s phenotypic values [15].

### Data analysis

We applied the NPBAT method to the Genetic Epidemiology of COPD (COPDGene) Study which is a multi-center case/control study designed to identify genetic factors associated with COPD and to characterize COPD-related phenotypes [19]. The study recruited COPD cases and smoking controls who were non-Hispanic whites and African Americans ages 45 to 80 with at least 10 pack-years of smoking history. The study also collected the Fagerstrom Test for Nicotine Dependence (FTND) to assess nicotine dependence, but the FTND score was only available for cases and controls who were current smokers at study enrollment. This data analysis represents the scenario where the secondary phenotype (FTND score) is available only in current smokers but the genotypic information is available for both current and former smokers. In the first 1,000 Non-Hispanic White (NHW) individuals, the FTND score controlling for age and gender was tested for an association with SNPs in the CHRNA 3/5 region for COPD cases and controls who are current smokers and association was found for rs1051730 or rs8034191 [20]. We applied the NPBAT statistic to the first 1000 NHW using the genotype of both current (307 individuals) and former smokers (669 individuals), controlling for age and gender and obtained the results shown in Table 1 for these 2 SNPs. Note that the NPBAT statistic performed better than both the Improved Score Test and the regression controlling for age and gender.

**Table 1:** **This table displays the p-values for the association between the Fagerstrom Test for Nicotine Dependence (FTND) and the markers listed above for the different statistical tests: the NPBAT where **$E_{x}={\bar{x}}_{c}$**is the genotypic mean of the current smokers, NPBAT where**$E_{x}={\bar{x}}_{f}$**is the genotypic mean of the former smokers, the Improved Score Test and a linear regression**

**Method**	**NPBAT:**${\text{E}}_{\text{x}}={\bar{\text{x}}}_{\text{c}}$	**NPBAT:**${\text{E}}_{\text{x}}={\bar{\text{x}}}_{\text{f}}$	**Improved Score Test**	**Regression**
rs1051730	0.00134	0.00138	0.00227	0.00259
rs8034191	0.00386	0.00391	0.00694	0.00744

## Results and discussion

NPBAT is a new statistical framework for population based genetic association tests that does not require making specific assumptions about the distribution of the phenotype. By conditioning on the phenotype, NPBAT is robust against violations of phenotypic model assumptions. The practical implications of NPBAT are demonstrated when applied to the COPDGene Study. FNDS, a measure of nicotine dependence, was assessed in current smokers that represent 31% of study participants in COPDGene. We analyzed SNPs shown to be associated with FNDS [20]. NPBAT identified the same SNPs as conventional methods but with slightly greater statistical significance than a linear regression for FNDS controlling for age and gender or the improved score test. Other examples of applications of NPBAT are 

1. when a sample is ascertained based on case/control status and the phenotype of interest is correlated with case status

2. in a cohort study in which prevalent cases are excluded (i.e. the classic epidemiologic cohort study) and the phenotype of interest is correlated with the disease of interest

3. a pharmacogenetics study using a randomized clinical trial when participants are ascertained based on the levels of the target of therapy

The broad application of NPBAT is to scenarios where samples are ascertained based on selection criteria that are correlated with the phenotype of interest.

## Conclusions

In conclusion, the key advantage that defines the attraction of the proposed approach is its robustness against model specification of the phenotypes. This enables extensions to different types of traits and the integration of complex statistical models for the phenotype. While, at the same time, the validity of the approach is not compromised by such generalization. Though the power is sensitive to the offset choice, NPBAT is valid regardless of the offset. As with all population-based association tests, population stratification can be a problem. Adjusting for known population sub-structure using principal components of ancestral informative markers (AIMs) or using genomic controls can reduce the impact of population stratification. The NPBAT software package which implements this method is detailed in the Appendix.

## Appendix

### Appendix A: Offset choice when Y is binary

The following considers the offset choice for the coded trait T when Y is binary. Assume the phenotype of interest is binary and the genotype of interest follows an additive model. Let *r*_0_, *r*_1_, and *r*_2_ denote the number of cases with 0, 1, and 2 disease alleles, respectively. Let *R* denote the total number of cases. Let *S* denote the total number of controls. Let *n*_0_, *n*_1_, and *n*_2_ denote the number of cases and controls with 0, 1, and 2 disease alleles, respectively. Let *N*=*S*+*R* denote the total number of cases and controls. In this scenario, the standard statistical method used is the Cochran-Armitage Trend test which can be written as follows:

(6) $$z_{\text{Cochran}}=\frac{N\left(r_{1}+2r_{2}\right)-R\left(n_{1}+2n_{2}\right)}{\sqrt{\left(\frac{\text{SR}}{N}\right)\left(N\left(n_{1}+4n_{2}\right)-{\left(n_{1}+2n_{2}\right)}^{2}\right)}}$$

In this scenario, let the coded phenotype *T*_*i*_ = *Y*_*i*_ - *μ*_*y*_ where *μ*_*y*_ is the offset. The NPBAT statistic has the following form:

(7) $$\;\frac{N\left(r_{1}+2r_{2}\right)-R\left(n_{1}+2n_{2}\right)}{\sqrt{\left(\left(\frac{N\mu_{y}^{2}}{R}\right)+\left(\frac{N{\left(1-\mu_{y}\right)}^{2}}{S}\right)\right)\left(\frac{\text{SR}\left(N(n_{1}+4n_{2})-{\left(n_{1}+2n_{2}\right)}^{2}\right)}{N-1}\right)}}$$

Note that the numerators of both statistics are the same. The ratio of the test statistics can be written as follows:

(8) $$\;\frac{\text{Sta}t_{\text{Cochran}}}{\text{Sta}t_{\text{NPBAT}}}=\sqrt{\frac{N}{N-1}}\sqrt{\left(1+\frac{1}{\gamma }\right)\mu_{y}^{2}+\left(1+\gamma \right){\left(1-\mu_{y}\right)}^{2}}$$

where $\gamma =\frac{\text{\#cases}}{\text{\#controls}}$. Given this ratio, the power of the NPBAT statistic relative to the Cochran-Armitage trend test is maximized for the offset choice $\mu_{y}^{\text{optimal}}=\frac{\gamma }{1+\gamma }=\frac{\text{\#cases}}{N}$. For example, if the ratio of the cases versus the controls is 1, the offset choice *μ*_*y*_ is $\frac{1}{2}$. This corresponds to equally weighting the cases and controls in the conditional test statistic. For large sample size N, such that $\sqrt{\frac{N}{N-1}}\approx 1$, the ratio of the test statistics is approximately one when the offset is set to $\mu_{y}^{\text{optimal}}=\frac{\text{\#cases}}{n}$. Consequently, for the optimal offset choice, the test statistics are approximately the same.

### Appendix B: asymptotic distribution when the secondary phenotype is available for both the cases and controls

To derive the asymptotic distribution of the NPBAT statistics for various phenotypic offset choices, let $\sigma_{X}^{2}$ denote the variance of X and $\sigma_{Y}^{2}$ denote the variance of Y. Let ||*a*|| denote the Euclidean norm. Let *T*_offset_ = ((*Y*_1_ - *Y*_offset_)...(*Y*_*n*_ - *Y*_offset_))^*t*^ and let $T_{\mu }={\left(T_{\mu_{1}},\mathrm{...},T_{\mu_{n}}\right)}^{t}={\left((Y_{1}-\bar{Y})\mathrm{...}(Y_{n}-\bar{Y})\right)}^{t}$ where $T_{\mu_{i}}=(Y_{i}-\bar{Y})$. Let $X^{t}=(X_{1}-\bar{X},\mathrm{...},X_{n}-\bar{X})$. Define $Z_{i}=\frac{(X_{i}-\bar{X})T_{\mu_{i}}}{||T_{\mu }||\hat{\sigma_{x}}}$. Then $\overset{n}{\underset{i=1}{\sum }}Z_{i}=\frac{X^{t}T_{\mu }}{||T_{\mu }||\hat{\sigma_{x}}}$. By treating X as random given Y is fixed, it can be shown that the *Z*_*i*_s are independent, *E*(*Z*_*i*_) = 0 and $\text{Var}\left(\overset{n}{\underset{i=1}{\sum }}Z_{i}\right)=1$. The Lindberg condition [25] for *Z*_*i*_, which ensures asymptotic normality of $\sum Z_{i}$, is then given by

(9) $$\forall \epsilon >0:\text{li}m_{n\to \infty }\left\{\overset{n}{\underset{i=1}{\sum }}\int_{|Z_{i}|\geq \epsilon }Z_{i}^{2}\text{dP}\right\}=0$$

Since *Z*_*i*_ has a discrete distribution, the Lindberg condition can only be fulfilled when the integration set {|*Z*_*i*_| ≥ *ϵ*} is empty for *n* → *∞*. Since X is the coded genotype and Y is a biological quantity, assume $\hat{\sigma_{x}}\neq 0$, $\hat{\sigma_{y}}\neq 0$ and both are finite. Then, there exists some constant K such that $\frac{\left|\left(X_{i}-\bar{X}\right)\right|\left|T_{\mu_{i}}\right|}{\hat{\sigma_{x}}\hat{\sigma_{y}}}\leq K$. Hence we rewrite the Lindberg condition by

(10) $$\;\forall \epsilon >0:\epsilon \leq \left|Z_{i}\right|=\frac{\left|\left(X_{i}-\bar{X}\right)\right|\left|T_{\mu_{i}}\right|}{\hat{\sigma_{x}}||T_{\mu }||}\leq \frac{K}{n}\to 0\;\text{as}\;n\to \infty$$

Hence the integral in the Lindberg condition is always computed over a set that is empty for *n* → *∞*. Thus the Lindberg condition is always fulfilled when the regularity condition holds. Then the Lindberg theorem [26] implies convergence to normality. Then

(11) $$\left(\frac{\left||T|\right|}{\left||T_{\mu }|\right|}\right)\text{Sta}t_{\text{NPBAT}}=\overset{n}{\underset{i=1}{\sum }}Z_{i}\to^{d}N\left(0,1\right)$$

Note that the statistic is maximized and has a standard normal distribution when *Y*_offset_ = *E*[*Y*].

### Appendix C: asymptotic distribution when the secondary phenotype is only available for the cases

Here, we derive the asymptotic distribution of the NPBAT statistic for secondary phenotypes in case/control studies. Consider a case control study where genetic information is available for both the cases and the controls, but the phenotypic information is only available for the cases. Here *n* is only the number of cases and all summations are only over the number of cases since the phenotypic information is not available for the controls where as in Appendix Appendix B: asymptotic distribution when the secondary phenotype is available for both the cases and controls, *n* is the number of cases and controls and the summation is over both the number of cases and controls. Let ${\bar{X}}_{\text{cases}}$ denote the sample mean of the genotypes of the cases and $\sigma_{X}^{2}$ be the true variance of the genotypes. Let $E_{x}={\bar{X}}_{\text{controls}}$ be the sample mean of the genotypes of the controls. Under the null hypothesis and assuming no population stratification, the sample mean of the genotypes of the cases and the sample mean of the genotypes of the controls both converge to *E*[*X*] since X is not associated with Y. Let $X_{\text{text}}={\left(X_{1}-{\bar{X}}_{\text{text}}\mathrm{...}X_{n}-{\bar{X}}_{\text{text}}\right)}^{t}$ where *text* = cases or controls, meaning *X*_1_..*X*_*n*_ is the coded genotype of the cases but $\bar{X}$ can be computed based on the cases, the controls, or both. Define

(12) $$Z_{i}=\frac{\left(X_{i}-{\bar{X}}_{\text{control}}\right)\left(Y_{i}-Y_{\text{offset}}\right)}{\hat{\sigma_{x}}\sqrt{||T_{\mu }|{|}^{2}+2{\left(\bar{Y}-Y_{\text{offset}}\right)}^{2}}}$$

then

(13) $$\begin{array}{ll} \overset{n}{\underset{i=1}{\sum }}Z_{i} & =\frac{X_{\text{control}}^{t}T}{\hat{\sigma_{x}}\sqrt{||T_{\mu }|{|}^{2}+2{\left(\bar{Y}-Y_{\text{offset}}\right)}^{2}}}\\ & =\frac{X_{\text{case}}^{t}T_{\mu }+n({\bar{X}}_{\text{case}}-{\bar{X}}_{\text{control}})(\bar{Y}-Y_{\text{offset}})}{\hat{\sigma_{x}}\sqrt{||T_{\mu }|{|}^{2}+2{\left(\bar{Y}-Y_{\text{offset}}\right)}^{2}}} \end{array}$$

It is important to note that the *Z*_*i*_s are independent, *E*(*Z*_*i*_) = 0 and $\text{Var}\left(\overset{n}{\underset{i=1}{\sum }}Z_{i}\right)=1$, which is obtained by first taking the conditional expectation treating X as random and Y as fixed. The Lindberg condition [25] for *Z*_*i*_, which ensures asymptotic normality of $\sum Z_{i}$, is then given by

(14) $$\forall \epsilon >0:\text{li}m_{n\to \infty }\left\{\overset{n}{\underset{i=1}{\sum }}\underset{|Z_{i}|\geq \epsilon }{\int }Z_{i}^{2}\text{dP}\right\}=0$$

Since *Z*_*i*_ has a discrete distribution, the Lindberg condition can only be fulfilled when the integration set {|*Z*_*i*_| ≥ *ϵ*} is empty for *n* → *∞*. Since X is the coded genotype and Y is a biological quantity, assume $\hat{\sigma_{x}}\neq 0$, $\hat{\sigma_{y}}\neq 0$ and both are finite. Then, there exists some constant K such that $\frac{\left|\left(X_{i}-{\bar{X}}_{\text{control}}\right)\right|\left|T_{i}\right|}{\hat{\sigma_{x}}\hat{\sigma_{y}}}\leq K$. Hence we rewrite the Lindberg condition by

(15) $$\begin{array}{ll} \forall \epsilon >0:\epsilon \leq \left|Z_{i}\right| & =\frac{\left|\left(X_{i}-{\bar{X}}_{\text{control}}\right)\right|\left|T_{i}\right|}{\hat{\sigma_{x}}\sqrt{||T_{\mu }|{|}^{2}+2{\left(\bar{Y}-Y_{\text{offset}}\right)}^{2}}}\\ & \leq \frac{\left|\left(X_{i}-{\bar{X}}_{\text{control}}\right)\right|\left|T_{i}\right|}{\hat{\sigma_{x}}||T_{\mu }||}\leq \frac{K}{n}\to 0\;\text{as}\;n\to \infty \end{array}$$

Hence the integral in the Lindberg condition is always computed over a set that is empty for *n* → *∞*. Thus the Lindberg condition is always fulfilled when the regularity condition holds. Then the Lindberg theorem [26] implies convergence to normality. Then

(16) $$\;\frac{\left||T|\right|}{\sqrt{||T_{\mu }|{|}^{2}+2{\left(\bar{Y}-Y_{\text{offset}}\right)}^{2}}}\text{Sta}t_{\text{NPBAT}}=\overset{n}{\underset{i=1}{\sum }}Z_{i}\to^{d}N(0,1)$$

Then the NPBAT statistic is normally distributed with mean zero and variance given above. Note that the variance is always greater than or equal to one and equals one when *Y*_offset_ = *E*[*Y*]. Note that if $Y_{\text{offset}}=\bar{Y}$ and $E_{x}={\bar{X}}_{\text{controls}}$ then NPBAT has a standard normal distribution. As seen in the Simulations section and Figure 1, when *E*_*x*_ is based on the the controls and the phenotype information is only available for the cases, then the power is maximized when $Y_{\text{offset}}\neq \bar{Y}$ because the variance equals the minimum when *Y*_offset_ ≈ *E*[*Y*].

### Appendix D: NPBAT software

A software package implemented in C++ to compute both single phenotype and multiple phenotypes NPBAT statistics is available for download at the following website: https://sites.google.com/site/genenpbat/. In addition to NPBAT statistics, other population based statistics such as the Armitage Trend Test, Fisher Exact Test are also available. Currently, only two platforms are supported: linux64 and windows64. The NPBAT software package reads in genetic data through the PLINK style pedigree (ped), map (map) and phenotype (phe) files. The website provides detail information on how to use the software package.

## Abbreviations

COPD: Chronic obstructive pulmonary disease; FBAT: Family Based Association Test; FEV: Forced expiratory volume; FNDS: Fagerstrom nicotine dependence score; GWAS: Genome Wide Association Study; LRT: Likelihood ratio test; NPBAT: Nonparametric Population Based Association Test; PC: Principal component.

## Competing interests

The authors declare that they have no competing interests.

## Authors’ contributions

SL derived the asymptotic distribution, performed the simulations studies and the data analysis. SL, WKY, JH, NL, and CL were involved in drafting the manuscript or revising it critically. CL made substantial contributions to conception of the method and assisted in the simulation studies. All authors read and approved the final manuscript.
